# Validation and Exploratory Refinement of the HFA-ICOS Score for Cardiovascular Risk in Proteasome Inhibitor-Treated Multiple Myeloma: Single-Center Retrospective Study

**DOI:** 10.3390/cancers18121924

**Published:** 2026-06-12

**Authors:** Eduardo Pons-Fuster, Alejandro Riquelme-Perez, Vyacheslav Shumbar, Celia María González-Ponce, Juan José Fernandez-Avila, Andrés Ramón-Martínez, María José Moreno-Belmonte, Valentín Cabañas-Perianes, Domingo Pascual-Figal, María Sacramento Diaz-Carrasco, Cesar Caro-Martínez

**Affiliations:** 1 Clinical Pharmacy and Therapeutics Research Group, Virgen de la Arrixaca University Clinical Hospital, Biomedical Research Institute of Murcia Pascual Parrilla (IMIB), 30120 Murcia, Spain; 2 Cardiology Department, Virgen de la Arrixaca University Clinical Hospital, Biomedical Research Institute of Murcia Pascual Parrilla (IMIB), 30120 Murcia, Spain; 3 Hematology Department, Virgen de la Arrixaca University Clinical Hospital, Biomedical Research Institute of Murcia Pascual Parrilla (IMIB), 30120 Murcia, Spain; 4 Medicine Department, University of Murcia, 30003 Madrid, Spain; 5 Centro Nacional de Investigaciones Cardiovasculares (CNIC), 28029 Madrid, Spain

**Keywords:** multiple myeloma, proteasome inhibitors, cardiovascular adverse events, NT-proBNP, HFA-ICOS risk score, cardio-oncology

## Abstract

**Background**: Proteasome inhibitors (PIs) are integral in multiple myeloma (MM) treatment but carry a substantial risk of cardiovascular adverse events (CVAEs). The Heart Failure Association–International Cardio-Oncology Society (HFA-ICOS) risk score was designed to identify patients at risk of cardiotoxicity, but its performance in MM remains uncertain. **Methods**: We retrospectively evaluated 98 patients with MM or primary amyloidosis treated with PIs between 2019 and 2024 to externally validate and refine this score. **Results**: CVAEs occurred in 22 patients (22.5%), predominantly heart failure. The original HFA-ICOS model demonstrated modest predictive accuracy (AUC 0.66) and sensitivity (50%), with frequent risk overclassification. Carfilzomib exposure (HR 4.68, 95% CI 1.47–14.90; *p* = 0.009) and elevated NT-proBNP before cycle 2 (>300 pg/mL; HR 3.13, 95% CI 1.10–8.93; *p* = 0.033) independently predicted CVAEs. A dynamic model incorporating these parameters and adjusting the age threshold to ≥65 years was associated with improved discrimination (AUC 0.72, *p* = 0.032), model fit (ΔAIC = −4), and CVAE-free survival stratification (*p* = 0.026), achieving 90.9% sensitivity. **Conclusions**: These findings indicate that the original HFA-ICOS score has limited prognostic value in PI-treated MM patients. Incorporating early NT-proBNP monitoring, carfilzomib exposure, and refined age categorization may improve risk prediction and support more personalized cardiovascular surveillance strategies in cardio-oncology. However, this refined dynamic model should be regarded as exploratory and requires validation in larger independent cohorts before it can be considered for clinical application.

## Simple Summary

Multiple myeloma is a type of blood cancer often treated with drugs called proteasome inhibitors. While these treatments are effective, they can increase the risk of heart problems such as heart failure or abnormal heart rhythms. Doctors use scoring tools to estimate which patients are at higher risk, but the accuracy of these tools in this setting is uncertain. In this study, we analyzed patients treated in a hospital to evaluate how well an existing risk score predicts heart complications. We found that the score had limited ability to correctly identify high-risk patients. We then explored a modified approach that includes checking a blood marker related to heart stress early during treatment, as well as considering specific drugs and patient age. This approach showed better ability to detect patients at risk. These findings suggest that early monitoring during treatment may help doctors better identify patients who need closer follow-up, although larger studies are needed to confirm these results.

## 1. Introduction

Cardiovascular toxicity is an increasing concern in cancer care, particularly with targeted therapies such as proteasome inhibitors (PIs). Widely used in hematologic malignancies [1,2], either alone or in combination with immunomodulatory drugs (IMiDs), proteasome inhibitors are associated with a significant risk of cardiovascular adverse events (CVAEs) [3,4,5,6,7,8,9,10]. Among them, carfilzomib has demonstrated a particularly unfavorable safety profile, with higher toxicity rates than bortezomib in both trials and observational studies [3,4,8,11]. As these agents become more common in clinical practice, early identification of patients at increased cardiovascular risk is essential to guide monitoring and preventive strategies.

There is growing recognition within cardio-oncology that structured cardiovascular risk assessment is essential to support informed clinical decision-making and improve patient care [12]. The Heart Failure Association (HFA) and International Cardio-Oncology Society (ICOS) have developed several drug-specific risk models for cardiotoxic anticancer agents, incorporating clinical history, comorbidities, biomarkers, and imaging findings [13]. While these tools represent an important step toward standardizing care, many, including the HFA-ICOS score for PIs and IMIDs in multiple myeloma, lack external validation, limiting their practical utility [14].

A major limitation of the current model is its reliance on baseline N-terminal pro-B-type natriuretic peptide (NT-proBNP), which is frequently elevated in cancer patients due to age, renal dysfunction, or comorbidities [15,16]. While markedly elevated NT-proBNP often reflects true cardiac stress, mild elevations may compromise the specificity of the tool and could lead to risk overestimation. Recent evidence suggests that NT-proBNP levels measured prior to cycle 2 of treatment (pre-cycle 2) more accurately reflect therapy-related cardiac stress and better predict subsequent CVAEs [8,17].

In this study, we aimed to externally validate the HFA-ICOS risk score for PIs and IMIDs in a real-world cohort of patients with multiple myeloma or primary amyloidosis. Furthermore, we evaluated whether selected modifications to the original score, including the incorporation of pre-cycle 2 NT-proBNP levels and other clinically relevant adjustments, could improve cardiovascular risk stratification.

## 2. Methods

### 2.1. Study Design and Patients

A retrospective, single-center study including patients treated with PIs between 2019 and 2024 was conducted. Patients were initially identified by search of the pharmacy dispensing database, and then electronic medical records were reviewed to confirm PI administration. All patients with MM or primary amyloidosis undergoing PI treatment (bortezomib or carfilzomib, either as a single agent or in combination therapy) initiated in our hospital were included. This study adhered to the Declaration of Helsinki and Good Clinical Practice Guidelines and was approved by the local Ethics Committee in April 2025 (2025-4-3-HCVUA).

### 2.2. Baseline Risk Factors and Patient Classification

The HFA-ICOS risk score for PIs and IMIDs for multiple myeloma [13] comprises six groups of variables: previous cardiovascular diseases (HF or cardiomyopathy, prior proteasome inhibitor or immunomodulatory drug cardiotoxicity, venous thrombosis [deep vein thrombosis or pulmonary embolism], cardiac AL amyloidosis, arterial vascular disease [Ischemic heart disease, percutaneous coronary intervention, coronary artery bypass graft, stable angina, transient ischaemic attack, stroke, peripheral vascular disease], baseline Left Ventricular Ejection Fraction [LVEF] < 50%, borderline LVEF 50–54%, arrhythmia [atrial fibrillation, atrial flutter, ventricular tachycardia or ventricular fibrillation] and left ventricular hypertrophy [left ventricular wall thickness > 1.2 cm]), cardiac biomarkers (elevated baseline troponin [>14 ng/L] and BNP or NT-proBNP [>125 pg/mL]), demographic and CV risk factors (age ≥75 years, age 65–74 years, hypertension [systolic blood pressure >140 mm Hg or diastolic BP > 90 mm Hg, or on treatment], diabetes mellitus, hyperlipidemia [non-HDL cholesterol level >145 mg/dL], chronic kidney disease [estimated glomerular filtration rate <60 mL/min/1.73 m^2^] and family history of thrombophilia), previous cardiotoxic cancer treatment (prior anthracycline exposure or thoracic spine radiotherapy), current myeloma treatment (high dose dexamethasone >160 mg/month) and lifestyle risk factors (current smoker or significant smoking history and obesity [BMI > 30]). The score established four categories based on future risk of cardiotoxicity (low, medium, high, and very high risk) (Supplementary Table S1). Additionally, other variables were collected to assess potential predictors of CVAEs.

### 2.3. Cardiovascular Adverse Events

CVAEs were defined as a patient having at least one of the following conditions after treatment with at least one dose of PIs: cardiovascular death, arrhythmias (atrial fibrillation/flutter), heart failure, worsening/new arterial hypertension and thromboembolic or ischemic events. All CVAEs were cardiologist-assessed and evaluated only while patients were on active therapy with proteasome inhibitor treatment. CVAEs were defined according to standardized criteria and consistent with current cardio-oncology recommendations [18]. Heart failure was defined according to the 2021 European Society of Cardiology Guidelines as the presence of typical symptoms and/or signs of heart failure in conjunction with objective evidence of cardiac structural or functional abnormalities. Objective evidence included a reduced left ventricular ejection fraction, signs of diastolic dysfunction on echocardiography, and/or elevated natriuretic peptide levels (NT-proBNP ≥ 125 pg/mL in stable patients or ≥300 pg/mL in acute) [19,20]. Arrhythmia was considered as new-onset or worsening atrial fibrillation or flutter confirmed by ECG. Hypertension was defined as sustained systolic blood pressure ≥140 mmHg or diastolic blood pressure ≥ 90 mmHg, or the need for new or intensified antihypertensive treatment. Ischemic or thromboembolic events included myocardial infarction, stroke, transient ischemic attack, or systemic arterial/venous thrombosis confirmed by imaging or biomarkers. Cardiovascular death was adjudicated when the treating team considered the primary cause of death to be cardiovascular, such as myocardial infarction, heart failure, arrhythmia, or stroke. For consistency with oncologic safety reporting standards, all events were additionally graded according to the Common Terminology Criteria for Adverse Events (CTCAE) v5.0 (Supplementary Table S2).

### 2.4. Study Procedures

Baseline high-sensitivity troponin T (hs-TnT) and NT-proBNP levels were analyzed at the central laboratory of the Virgen de la Arrixaca University Clinical Hospital (Cobas^®^ 8000 system, Roche Diagnostics, Mannheim, Germany) using commercially available electrochemiluminescence immunoassays (Elecsys proBNP II and Troponin T hs, Roche Diagnostics). Additionally, comprehensive lab tests including NT-proBNP levels were recorded pre-cycle 2 (approximately one month following the initial PI dose and prior to the second treatment cycle) to evaluate changes in the cardiac biomarker. While the HFA-ICOS score defines elevated NT-proBNP as a value above the upper limit of the local laboratory reference range (>125 pg/mL), a higher cutoff of >300 pg/mL was also applied, based on prior research [19,20]. Transthoracic echocardiography was performed by experienced cardiologists at a single tertiary center. All examinations followed current recommendations for the echocardiographic assessment of cardiac structure and function in adults, ensuring consistency in image acquisition and interpretation [21].

### 2.5. Development of Refined HFA-ICOS Models

The performance of the original HFA-ICOS score was assessed, and refined approaches were explored to improve cardiovascular risk stratification in this population. A first refined model was defined through targeted modifications to the original score, informed by existing literature, clinical rationale, and observations derived from the study cohort. These modifications included: (i) the application of a higher NT-proBNP threshold (>300 pg/mL instead of >125 pg/mL at baseline), based on prior literature and guideline recommendations supporting this cutoff as more specific for clinically relevant cardiac dysfunction [19,20], (ii) the incorporation of carfilzomib exposure as a high-risk variable, consistent with previous reports describing higher cardiotoxicity associated with carfilzomib [8,11], and (iii) the reclassification of age ≥65 years as a medium-risk factor. A second model incorporating early on-treatment information (hereafter referred to as the “dynamic” model) was subsequently evaluated. In this model, baseline NT-proBNP was replaced by pre-cycle 2 NT-proBNP values, while maintaining these modifications. Both refined models were evaluated alongside the original HFA-ICOS score to examine their potential to improve predictive performance.

### 2.6. Statistical Analysis

Statistical analyses were conducted using R software (v4.4.1; R Core Team, Vienna, Austria, 2024). Descriptive statistics were employed to summarize the data: categorical variables were reported as frequencies and percentages, while continuous variables were expressed as mean and standard deviation (SD), or alternatively, median with interquartile range [Q1–Q3], depending on their distribution. The Shapiro–Wilk test was used to assess the normality of continuous variables. Group comparisons between patients with and without CVAEs were performed using Fisher’s exact test for categorical variables, and the Mann–Whitney U test or Kruskal–Wallis test for non-normally distributed continuous variables. Bonferroni correction was applied to adjust for multiple comparisons. The discriminative performance of the HFA-ICOS score was evaluated using receiver operating characteristic (ROC) curves, and the area under the curve (AUC), which was calculated to quantify its discriminatory ability. Calibration was assessed using the Brier score, calibration intercept and slope, and the Hosmer–Lemeshow goodness-of-fit test to evaluate the agreement between predicted and observed event probabilities. Comparisons between ROC curves for the conventional and refined scores were performed using the DeLong test to assess differences in AUCs. Model fit was compared using the Akaike Information Criterion (AIC), with lower AIC values indicating better model performance. Kaplan–Meier survival curves were generated to assess differences in CVAE-free survival across HFA-ICOS risk categories, and comparisons were made using the log-rank test. To identify potential predictors of CVAEs, univariate and multivariate Cox proportional hazards regression models were fitted. Hazard ratios (HRs) and corresponding 95% confidence intervals (CIs) were reported for variables associated with the risk of developing CVAEs. Variables included in the multivariable model were selected based on pre-specified clinical relevance and statistical significance in univariable analysis. Cases with missing data were excluded from the corresponding analyses (complete-case analysis). Missing data were minimal across variables, and no imputation methods were applied. Statistical significance was defined as a two-sided *p*-value < 0.05.

## 3. Results

### 3.1. Participants’ Baseline Parameters, CV Risk Factors and Score Classification

**Figure 1:**
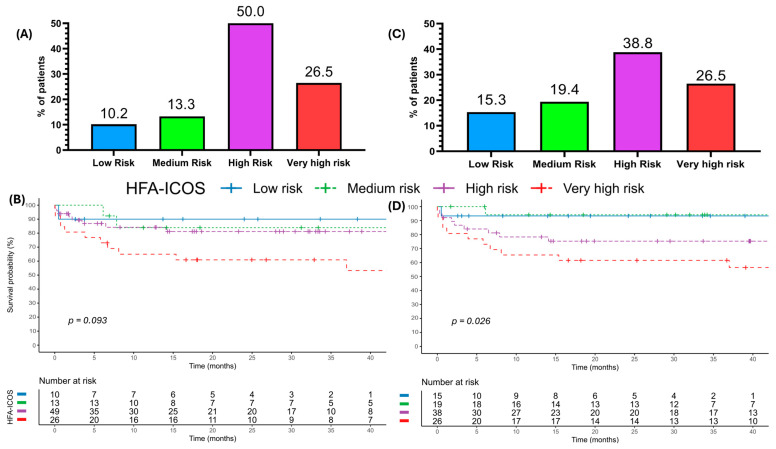
(**A**) Risk distributions and (**B**) event-free survival of the HFA-ICOS score and (**C**) risk distributions and (**D**) event-free survival of the refined (dynamic) HFA-ICOS score. Abbreviations: CVAE = cardiovascular adverse event; HFA-ICOS = Heart Failure Association–International Cardio-Oncology Society. Risk categories are color-coded as follows: low risk (blue), medium risk (green), high risk (purple), and very high risk (red).

A total of 120 patients treated with proteasome inhibitors (PIs) were identified in our medical databases, of which 98 were included in the analysis after excluding those treated for conditions other than multiple myeloma or primary amyloidosis (Supplementary Figure S1). Oncological and pharmacological baseline variables of the whole cohort are summarized in Table 1. The median duration of treatment was 5.4 months (IQR: 3.9–11.0). Twenty-two (22.5%) patients experienced CVAEs, and their associated risk factors based on the HFA-ICOS score can be observed in Table 2. Briefly, patients experiencing CVAEs were more frequently distributed in the 65–74 years group (45.5% vs. 22.4%, *p* = 0.033), had higher incidence of previous heart failure (22.7% vs. 6.6%, *p* = 0.028), cardiac AL amyloidosis (9.1% vs. 0.0%, *p* = 0.008), arrhythmia (30.0% vs. 7.7%, *p* = 0.007) and pre-cycle 2 NT-proBNP levels > 300 pg/mL (50.0% vs. 19.7%, *p* = 0.012). Additionally, a higher prevalence of CVAEs were observed in carfilzomib-treated patients compared to bortezomib (45.5% vs. 15.8%, *p* = 0.003). Absolute cardiac biomarker levels and cardiovascular treatment at baseline can be found in Supplementary Table S3 and Supplementary Table S4, respectively. Following the HFA-ICOS score, 10.2% of patients were classified as low-risk, 13.3% as medium-risk, 50.0% as high-risk and 26.5% as very high-risk (Figure 1A), of which a higher percentage belonged to the patients who experienced CVAEs (50.0% vs. 19.7%, *p* = 0.005).

### 3.2. CVAEs

**Figure 2:**
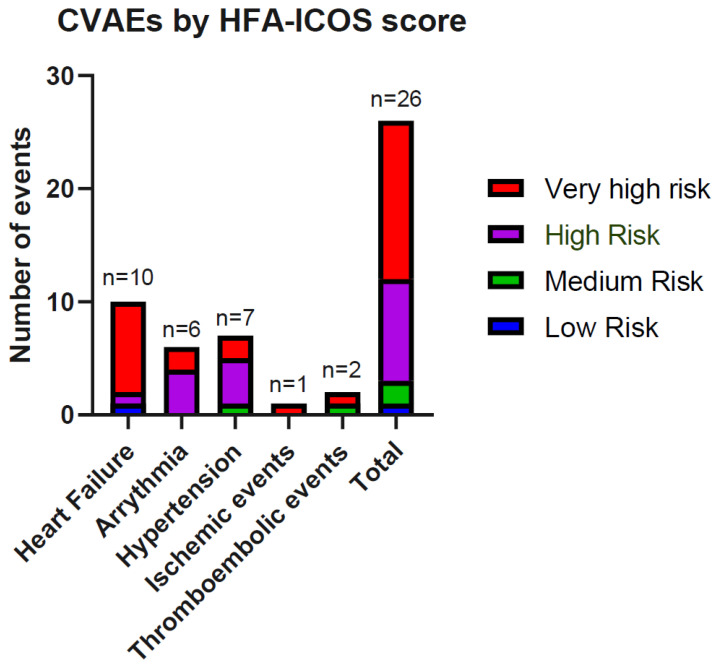
Cardiovascular adverse events (CVAEs) classified by the HFA-ICOS risk category. CVAEs included heart failure, arrhythmias, hypertension, ischemic events, and thromboembolic events. Abbreviations: CVAE = cardiovascular adverse event; HFA-ICOS = Heart Failure Association–International Cardio-Oncology Society.

Twenty-two patients experienced CVAEs (22.5%) and a total of 26 CVAEs were registered, 16 classified as grade 1–2 (mild to moderate) and 10 as grade 3–4 (severe to life-threatening) (Supplementary Table S5). Median time to event was 2.8 months (range, 0.1–49.3 months). Heart failure was the most frequently observed CVAE (10.2%), followed by worsening/new arterial hypertension (7.1%), arrhythmia (6.1%), thromboembolic events (2.0%) and ischemic events (1.0%), while hypertension was the most frequent event graded as level 3–4 in the CTCAE scale (4.1%) (Figure 2).

### 3.3. HFA-ICOS Risk Prediction Model

**Figure 3:**
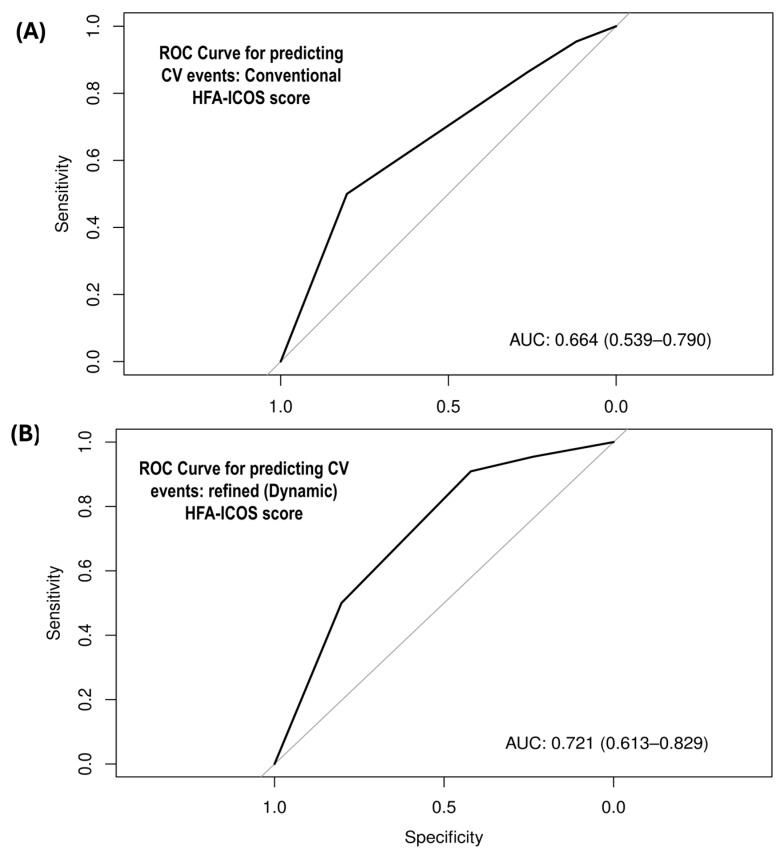
ROC Curve for predicting CV events for the (**A**) conventional and (**B**) dynamic HFA-ICOS score. Abbreviations: AUC = area under the curve; CV = cardiovascular; HFA-ICOS = Heart Failure Association–International Cardio-Oncology Society; ROC = receiver operating characteristic.

Performance tests showed that the HFA-ICOS risk stratification score had a 50.0% sensitivity, 80.3% specificity, 42.3% positive predictive value, 84.7% negative predictive value and 73.5% global accuracy precision when predicting the development of CVAEs. The HFA-ICOS risk stratification score demonstrated limited discrimination with an AUC of 0.66 (95% CI 0.54–0.79) for predicting CVAEs (Figure 3A) and modest calibration (Brier = 0.170; calibration intercept = 0.04, slope = 0.86; Hosmer–Lemeshow: *p* = 0.39). Kaplan–Meier survival curves for the CVAE stratified by HFA-ICOS are shown in Figure 1B, where no statistically significant differences are observed between the risk groups (log-rank *p* = 0.093).

### 3.4. Clinical Predictors of CVAEs

Univariate and multivariate Cox regression analyses were conducted to assess clinical factors that could predict the development of CVAEs in PI-treated patients (Table 3). In the univariate analysis, the following variables were significant: arrhythmia (HR 2.66, 95% CI 1.04–6.81; *p* = 0.041), previous cardiovascular disease (HR 2.48, 95% CI 1.08–6.72; *p* = 0.025), elevated baseline hs-TnT (HR 2.27, 95% CI 1.08–5.49; *p* = 0.035), carfilzomib vs. bortezomib (HR 3.45, 95% CI 1.49–8.00; *p* = 0.004), and elevation of pre-cycle 2 NT-proBNP > 300 pg/mL (HR 3.25, 95% CI 1.30–8.11; *p* = 0.012). In the multivariate analysis, both treatment with carfilzomib (HR 4.68, 95% CI 1.47–14.90; *p* = 0.009) and elevation of pre-cycle 2 NT-proBNP > 300 pg/mL (HR 3.13, 95% CI 1.10–8.93; *p* = 0.033) remained statistically significant.

### 3.5. Alternative HFA-ICOS Stratification Scores

A total of 61.2% and 24.5% of patients included in the study had baseline NT-proBNP levels > 125 pg/mL and were ≥75 years old, respectively, two high-risk variables that, under the conventional HFA-ICOS score, would classify patients directly into the high-risk group. To explore potential improvements in event prediction, we first evaluated a refined, baseline-only version of the HFA-ICOS model incorporating: (1) a higher cutoff for baseline NT-proBNP (300 pg/mL instead of 125 pg/mL); (2) carfilzomib exposure as a high-risk variable; and (3) reclassification of age ≥ 65 as medium risk (in the original HFA-ICOS score, age ≥ 75 years is classified as very high risk, and 65–74 years as medium risk). This model modestly improved discrimination compared with the original baseline model (AUC = 0.71 [95% CI 0.59–0.82], ΔAIC = −3.3) and calibration (Brier = 0.165; intercept = 0.02, slope = 0.91; Hosmer–Lemeshow: *p* = 0.53). However, the difference did not reach statistical significance using DeLong’s test (*p* = 0.078).

A model incorporating early on-treatment information (dynamic model) was subsequently evaluated by replacing baseline NT-proBNP with pre-cycle 2 NT-proBNP levels. The model incorporated: (1) pre-cycle 2 NT-proBNP (>300 pg/mL); (2) carfilzomib exposure as a high-risk variable; and (3) reclassification of age ≥ 65 as medium risk. Following this model, 15.3% of patients were classified as low-risk, 19.4% as medium-risk, 38.8% as high-risk and 26.5% as very high-risk (Figure 1C). Performance tests showed that the dynamic HFA-ICOS risk stratification score had a 90.9% sensitivity, 42.1% specificity, 31.3% positive predictive value, 94.1% negative predictive value and a 53.1% global accuracy when predicting the development of CVAEs. The dynamic HFA-ICOS risk model showed acceptable discrimination (AUC = 0.72 [95% CI 0.60–0.84]) (Figure 3B) and calibration (Brier = 0.152; intercept = 0.00, slope = 0.99; Hosmer–Lemeshow: *p* = 0.72). Kaplan–Meier survival curves showed significant differences between risk groups (log-rank *p* = 0.026). Kaplan–Meier survival curves for the CVAE stratified by dynamic HFA-ICOS are shown in Figure 1D, where statistically significant differences are observed between the risk groups (log-rank *p* = 0.026). When compared with the original score, the model was associated with a lower Akaike Information Criterion (ΔAIC = −4). DeLong’s test showed a statistically significant difference between models (*p* = 0.032).

## 4. Discussion

This study represents the first external validation of the HFA-ICOS cardiovascular risk assessment in patients with MM initiating PI therapy. While the original model demonstrated acceptable specificity, its limited sensitivity and overrepresentation in higher risk categories may reduce its clinical utility. However, given its retrospective design and relatively limited sample size, these findings should be interpreted with caution. In this context, we explored a refined approach incorporating pre-cycle 2 NT-proBNP levels, carfilzomib exposure as a high-risk factor, and reclassification of age ≥ 65 years to medium risk. Operationally, we propose a two-step workflow: (1) refined baseline HFA-ICOS classification and (2) reclassification of patients who are low/intermediate at baseline to high risk if pre-cycle-2 NT-proBNP ≥ 300 pg/mL. This approach does not replace baseline risk stratification but rather complements it by incorporating early on-treatment information to refine cardiovascular risk assessment after therapy initiation. This early-treatment refinement may help prioritize monitoring resources for patients who ‘convert’ to high risk after initial exposure.

Our cohort was predominantly composed of older patients with a high prevalence of cardiovascular comorbidities such as chronic kidney disease, diabetes, and prior heart disease. Bortezomib was the most frequently used proteasome inhibitor, while carfilzomib was administered in a smaller proportion of cases. During follow-up, approximately 25% of patients developed CVAEs, a similar rate to that reported in previous studies [4,7,9], highlighting the importance of implementing effective preventive strategies in this high-risk population. To better understand the predictors of CVAEs in this setting, we explored potential risk factors through univariate and multivariate analyses. In the univariate analysis, several variables showed a clear association with event occurrence, including arrhythmia, previous cardiovascular disease, carfilzomib use, elevated baseline troponin, and pre-cycle 2 NT-proBNP levels above 300 pg/mL. However, only carfilzomib use and pre-cycle 2 NT-proBNP levels above 300 pg/mL were associated with CVAEs in the multivariate analysis. This pattern aligns with previous studies, supporting the role of both clinical history and early biomarker changes as potential predictors of cardiotoxicity in patients receiving PIs [3,4,8,9,10].

Following the conventional HFA-ICOS score, more than three-quarters of patients were classified into the high- or very high-risk groups. Importantly, being classified into these risk groups does not mean that patients cannot receive their malignancy treatment; rather, it indicates the need for closer surveillance and early concomitant cardiac management. However, this imbalanced distribution likely overestimates baseline risk and limits the model’s ability to differentiate between individuals who genuinely need intensified cardiology follow-up and those who do not. Furthermore, its discriminative capacity was modest, with limited sensitivity. The absence of a clear gradient of event incidence across risk categories further undermines the model’s clinical utility in guiding cardiologic surveillance and treatment decisions. These limitations become more evident when compared to the performance of HFA-ICOS risk assessments for other cardiotoxic anticancer drugs. The anthracycline-specific score, recently validated in the CARDIOTOX registry, demonstrated a well-balanced risk distribution, with over 50% of patients categorized as low risk, and solid discriminative ability, with an AUC of 0.78 and a clear escalation in event incidence across increasing risk strata [22]. In contrast, validations of the anti-HER2 score have shown more modest performance. While the anti-HER2 tool showed an AUC of 0.64, most events occurred in the very high-risk group, with signs of overclassification in intermediate categories [23]. These findings suggest that, unlike the anthracycline risk calculators, the PIs and IMiDs as well as the anti-HER2 assessments have important limitations and support the need for further refinement.

Expert consensus has highlighted this inconsistency as a key gap in care and called for systematic strategies to improve baseline risk stratification [18,24]. There is a growing need to validate existing risk models in real-world settings and refine them to better predict cardiovascular toxicity based on drug- and patient-specific factors [14,25]. In the context of the limited performance observed for the original HFA-ICOS score in our cohort, several aspects of cardiovascular risk assessment may warrant further exploration. One such area of refinement is the use of natriuretic peptides. In patients with cancer, baseline NT-proBNP levels frequently show moderate elevations that do not necessarily reflect elevated intracardiac pressures, which limits the specificity of the biomarker in this context [26]. This has prompted the use of higher thresholds to enhance risk stratification, and a cutoff of 300 pg/mL has been supported by clinical guidelines and large-scale studies, where it has shown consistent performance and a high negative predictive value, even in older patients [19,20]. Importantly, prior studies in patients with multiple myeloma treated with PIs have shown that NT-proBNP measured prior to the second cycle is more predictive of cardiovascular events than baseline values [8,17], as also observed in our study. In our cohort, NT-proBNP was measured after the first treatment cycle (approximately one month after initiation and before the 2nd treatment cycle), and most CVAEs occurred after this timepoint. Of the 22 CVAEs identified, 5 occurred before the pre-cycle 2 NT-proBNP assessment. These patients had already been classified as very high risk at baseline, and therefore the biomarker measurement did not alter their risk categorization. Consequently, in the remaining 17 patients, elevated NT-proBNP levels preceded the clinical event. Interestingly, the proportion of patients with NT-proBNP > 300 pg/mL was less frequent at pre-cycle 2 than at baseline, which may reflect early clinical optimization or biological variability, although values remained higher in patients who developed CVAEs. In our exploratory analyses, incorporating pre-cycle 2 NT-proBNP was associated with improved discrimination compared to baseline-only approaches, supporting the potential value of early on-treatment biomarker reassessment.

In the context of these limitations, the differential cardiotoxicity of individual proteasome inhibitors may represent an additional factor to consider. Despite growing evidence of increased cardiotoxicity with carfilzomib relative to other PIs, the existing HFA-ICOS score does not account for differences between individual agents. In our exploratory analyses, the inclusion of carfilzomib as a high-risk variable was supported by several considerations: a significant proportion of patients who experienced CVAEs in our cohort were treated with carfilzomib; it was independently associated with CVAEs in multivariate analysis; and previous studies have consistently reported higher cardiotoxicity with carfilzomib compared to other PIs [3,4,8,11]. Underlying mechanisms such as endothelial dysfunction, increased oxidative stress, and mitochondrial impairment as well as irreversible proteasome inhibition could act as potential contributors to its elevated cardiovascular risk [3,6,27]. Similarly, age reclassification was explored based on both our findings and limited supporting evidence. In this approach, age was treated as a single medium-risk variable (≥65 years), effectively removing the additional weighting assigned to patients aged ≥ 75 years in the original score. While age is a well-established cardiovascular risk factor in the general population, its predictive value for treatment-related CVAEs appears to be limited. Notably, its inclusion as a high-risk variable in the original HFA-ICOS score is supported only by level C evidence, and most studies have not identified age as an independent predictor once other variables are considered [4,5,7,8,11,28,29]. These considerations suggest that age ≥ 75 years alone may not warrant classification as a high-risk factor in this specific context.

Considering the limited performance of the original HFA-ICOS score in this cohort, our exploratory analysis suggests that incorporating pre-cycle 2 NT-proBNP, carfilzomib as a high-risk variable and reclassification of age ≥ 65 years to medium risk may contribute to improved risk stratification and could offer additional clinical value by capturing early subclinical cardiac stress. This adjustment was associated with increased sensitivity and negative predictive value, while also reducing the proportion of patients categorized as high risk. While these findings should be interpreted with caution, the dynamic approach allows for a more refined classification of cardiovascular risk. In a cardio-oncology setting, where early identification of high-risk patients is important for guiding clinical decisions, this approach could help inform the intensity of monitoring strategies.

This study has several limitations that should be acknowledged. Its retrospective, single-center design and modest sample size (22 events among 98 patients; EPV ≈ 3.7) may limit generalizability and raise the risk of overfitting. Therefore, the multivariable findings should be considered exploratory, and internal validation was not feasible due to the limited number of events, although model calibration was assessed, supporting discrimination results. A small number of patients had primary amyloidosis, which may introduce some degree of clinical heterogeneity and potentially influence baseline cardiovascular risk. There was heterogeneity in the type of proteasome inhibitors and treatment regimens, which could have influenced cardiovascular risk and model performance across subgroups. Although NT-proBNP levels were consistently measured, the analysis focused mainly on early-phase assessments and did not explore the prognostic value of longer-term monitoring. Although implementation may still vary across centers, routine pre-cycle 2 NT-proBNP testing appears feasible given its broad availability and recommendation in cardio-oncology guidelines. Although our analysis focused on cardiovascular events occurring during active therapy, long-term cardiovascular toxicity remains an important challenge in cardio-oncology, particularly in patients with increasing life expectancy. This approach may not fully capture longer-term cardiovascular risk, and in this context, dynamic strategies may be relevant for longitudinal risk assessment. Nevertheless, most PI-related complications occur early after treatment initiation, and causal attribution becomes less reliable once therapy is modified or completed. Concomitant treatments may also have contributed to cardiotoxicity, while co-medications with cardioprotective effects, such as SGLT2 inhibitors [30,31], were underrepresented, despite growing evidence of their potential role in cardio-oncology. Another limitation is that the dynamic model, while improving sensitivity and event prediction, showed lower specificity than the conventional HFA-ICOS score, which could increase false positives and resource utilization by triggering additional cardiology evaluations. Nevertheless, it classified fewer patients as high risk and demonstrated a high negative predictive value, supporting its utility in identifying patients unlikely to require intensive monitoring. In cardio-oncology, where the consequences of missed risk are greater than those of overestimation, such a model may offer greater clinical value despite reduced specificity. Finally, the absence of an external validation of the refined model restricts generalizability, and confirmation in larger, multicenter cohorts will be essential to confirm model stability and generalizability. Despite these limitations, our results highlight important limitations in the current HFA-ICOS score and support the potential role of exploratory refinements in enhancing cardiovascular risk stratification in this setting.

## 5. Conclusions

This study provides the first external validation of the HFA-ICOS cardiovascular risk assessment for PI therapy in patients with MM. While the original score offers acceptable specificity, its limited sensitivity and tendency to overclassify baseline risk suggest that its clinical utility may be constrained in this setting. In this cohort, an exploratory approach incorporating pre-cycle 2 NT-proBNP levels and selected variable refinements was associated with improved risk discrimination, which could help inform more individualized cardiology follow-up strategies. These findings support the potential value of a more dynamic and personalized approach to cardiovascular risk stratification, integrating early on-treatment information to refine patient monitoring. However, this refined dynamic model should be regarded as exploratory and requires validation in larger, independent cohorts before it can be considered for clinical application.

## Figures and Tables

**Table 1:** Oncological and Pharmacological Baseline Variables.

Baseline Variables *n* (%)	All Patients (n = 98)	Cardiovascular Events (n = 22)	Non-Cardiovascular Events (n = 76)	*p*
Neoplasm				
Previous neoplasm	23 (23.5)	3 (13.6)	20 (26.3)	0.22
Current neoplasm, Multiple myeloma	94 (96.0)	20 (90.9)	74 (97.4)	0.18
Primary amyloidosis	4 (4.0)	2 (9.1)	2 (2.6)	
ECOG ≤ 1,	77 (78.6)	17 (77.3)	60 (78.9)	0.76
Previous therapies				
Number of prior treatment lines, 0	64 (65.3)	10 (45.5)	54 (71.1)	0.076
1	24 (24.5)	9 (40.9)	15 (19.7)	
≥2	10 (10.2)	3 (13.6)	7 (9.2)	
Prior chemotherapy	37 (37.8)	11 (50.0)	26 (34.2)	0.18
Prior proteasome inhibitor	26 (26.5)	10 (45.5)	16 (21.1)	0.022
Treatment regimen				
Dara-VRD	14 (14.3)	3 (13.6)	11 (14.5)	0.12
Krd	13 (13.3)	6 (27.3)	7 (9.2)	
VRd	19 (19.4)	2 (9.1)	17 (22.4)	
Other	52 (53.1)	11 (50.0)	41 (53.9)	
Combination therapy drugs				
Immunomodulatory drugs	72 (73.5)	15 (68.2)	57 (75.0)	0.34
Lenalidomide	54 (55.1)	12 (54.5)	42 (55.3)	0.95
Daratumumab	32 (32.7)	7 (31.8)	25 (32.9)	0.92
Treatment Follow-up				
Follow-up time, months, median [IQR]	26.3 [12.2–44.9]	24.2 [11.0–47.6]	27.4 [13.5–44.3]	0.93
Duration of treatment, months, median [IQR]	5.4 [3.9–11.0]	6.2 [3.9–14.7]	5.2 [4.0–11.0]	0.51
Treatment interruption	24 (24.5)	8 (36.4)	16 (21.1)	0.14
Dose reduction	49 (50.0)	10 (45.5)	39 (51.3)	0.63
Treatment discontinuation	31 (31.6)	9 (40.9)	22 (28.9)	0.29
Disease progression	28 (28.6)	6 (27.3)	22 (28.9)	0.88
Death	29 (29.6)	7 (31.8)	22 (28.9)	0.78

Abbreviations: ECOG, Eastern Cooperative Oncology Group performance status; Dara-VRD, daratumumab plus bortezomib, lenalidomide, and dexamethasone; Krd, carfilzomib, lenalidomide, and dexamethasone; VRd, bortezomib, lenalidomide, and dexamethasone; IQR, interquartile range.

**Table 2:** Patient Characteristics, Cardiovascular Risk Factors, and Risk Classification According to the HFA-ICOS Score.

Risk Factor, n (%)	All Patients (n = 98)	Cardiovascular Events (n = 22)	Non-Cardiovascular Events (n = 76)	*p*
Demographic and CV risk factors				
Sex, male	58 (59.2)	12 (54.4)	46 (60.5)	0.62
Age, years, mean ± SD	66.7 ± 11.5	68.5 ± 10.5	64.7 ± 11.8	0.18
Age ≥ 75 years	24 (24.5)	5 (22.7)	19 (25.0)	0.83
Age 65–74 years	27 (27.6)	10 (45.5)	17 (22.4)	0.033
Hypertension	51 (52.0)	14 (63.6)	37 (48.7)	0.21
Diabetes mellitus	21 (21.4)	2 (9.1)	19 (25.0)	0.11
Hyperlipidaemia	41 (41.8)	8 (36.4)	33 (43.4)	0.56
Chronic kidney disease	40 (40.8)	11 (50.0)	29 (38.2)	0.32
Creatinine mg/dL, median [IQR]	1.0 [0.7–1.7]	1.1 [0.8–1.9]	0.9 [0.7–1.6]	0.25
Family history of thrombophilia	0 (0.0)	0 (0.0)	0 (0.0)	0.99
Lifestyle risk factors				
Current smoker or smoking history	38 (38.8)	10 (45.5)	28 (36.8)	0.47
Obesity (BMI > 30)	28 (28.6)	7 (31.8)	21 (27.6)	0.70
Previous cardiovascular disease				
Heart failure or cardiomyopathy	10 (10.2)	5 (22.7)	5 (6.6)	0.028
Prior proteasome inhibitor cardiotoxicity	0 (0.0)	0 (0.0)	0 (0.0)	0.99
Venous thrombosis (DVT or PE)	6 (6.1)	1 (4.5)	5 (6.6)	0.73
Cardiac amyloidosis	2 (2.0)	2 (9.1)	0 (0.0)	0.008
Arterial vascular disease	1 (1.0)	1 (4.5)	0 (0.0)	0.062
Prior Immunomodulatory drug CV toxicity	0 (0.0)	0 (0.0)	0 (0.0)	0.99
Baseline LVEF ≤ 50	3 (3.1)	1 (4.5)	2 (2.6)	0.71
Borderline LVEF 50–54	4 (4.1)	1 (4.5)	3 (3.9)	0.97
Arrhythmia	12 (12.2)	6 (27.3)	6 (7.9)	0.015
Left ventricular hypertrophy	16 (16.3)	4 (18.2)	12 (15.8)	0.91
Cardiac biomarkers				
Baseline troponin (hs-TnT) > 14 pg/mL	39 (39.8)	13 (59.1)	26 (34.2)	0.080
NT-proBNP > 125 pg/mL	60 (61.2)	15 (68.2)	45 (59.2)	0.55
NT-proBNP > 300 pg/mL	33 (33.7)	10 (45.5)	23 (30.3)	0.22
NT-proBNP > 300 pg/mL pre-cycle 2	26 (26.5)	11 (50.0)	15 (19.7)	0.012
Previous cardiotoxic cancer treatment				
Prior anthracycline exposure	9 (9.2)	1 (4.5)	8 (10.5)	0.39
Prior thoracic spine radiotherapy	6 (6.1)	0 (0.0)	6 (7.9)	0.17
Current treatment				
Bortezomib	76 (77.6)	12 (54.5)	64 (84.2)	0.003
Carfilzomib	22 (22.4)	10 (45.5)	12 (15.8)	
High-dose dexamethasone > 160 mg/month	17 (17.3)	4 (18.2)	13 (17.1)	0.91
Risk level				
Low risk	10 (10.2)	1 (4.5)	9 (11.8)	0.32
Medium risk	13 (13.3)	2 (9.1)	11 (14.5)	0.51
High risk	49 (50.0)	8 (36.4)	41 (53.9)	0.15
Very high risk	26 (26.5)	11 (50.0)	15 (19.7)	0.005

Risk Category Definitions: Low risk = no risk factor OR one medium^1^-risk factor; Medium risk = medium-risk factors with a total of 2–4 points; High risk = medium-risk factors with a total of ≥5 points OR any high-risk factor; Very high risk = any very high-risk factor. Arrhythmia = Atrial fibrillation, atrial flutter, ventricular tachycardia or ventricular fibrillation; BMI = Body mass index; Chronic kidney disease = Estimated glomerular filtration rate < 60 mL/min/1.73 m^**2**^; CV = Cardiovascular disease; DVT = Deep vein thrombosis; hs-TnT = High-sensitivity Troponin T; Hyperlipidaemia = Non-HDL cholesterol level > 3.8 mmol/L (>145 mg/dL); Hypertension = Systolic blood pressure (BP) > 140 mmHg or diastolic BP > 90 mmHg, or on treatment; Left ventricular hypertrophy = Left ventricular wall thickness > 1.2 cm; LVEF = Left ventricular ejection fraction; NT-proBNP = N-terminal pro–B-type natriuretic peptide; PE = Pulmonary embolism.

**Table 3:** Univariate and multivariate Cox regression analysis of selected variables for predicting cardiovascular adverse events (CVAEs).

	Univariate		Multivariate	
	HR (95% CI)	*p*	HR (95% CI)	*p*
Age, years	1.02 (0.98–1.05)	0.34	-	-
≥2 CV risk factors	0.99 (0.42–2.32)	0.98	-	-
Chronic kidney disease	1.44 (0.62–3.32)	0.39	-	-
Previous CV disease	2.48 (1.08–6.72)	0.025	0.94 (0.26–3.43)	0.92
Arrythmia	2.66 (1.04–6.81)	0.041	2.29 (0.71–7.35)	0.16
Heart failure	1.89 (0.44–8.11)	0.39	-	-
Valvular heart disease	1.50 (0.35–6.46)	0.59	-	-
Elevated hs-TnT	2.27 (1.08–5.49)	0.035	1.41 (0.36–5.47)	0.62
Carfilzomib vs. bortezomib	3.45 (1.49–8.00)	0.004	4.68 (1.47–14.9)	0.009
Baseline NT-proBNP > 300	1.88 (0.80–4.43)	0.15	-	-
Pre-cycle 2 Nt-proBNP > 300	3.25 (1.30–8.11)	0.012	3.13 (1.10–8.93)	0.033

Abbreviations: CI: Confidence Interval; CV: cardiovascular; hs-TnT = High-sensitivity Troponin T; HR: Hazard Ratio; NT-proBNP = N-terminal pro–B-type natriuretic peptide.

## Data Availability

The data that support the findings of this study are available from the corresponding author upon reasonable request.

## Supplementary Materials

The following supporting information can be downloaded at https://www.mdpi.com/article/10.3390/cancers18121924/s1. Figure S1: Flow diagram; Table S1: Baseline cardiovascular risk stratification scale for proteasome inhibitors and immunomodulatory agents for multiple myeloma; Table S2: Definitions and grading of cardiovascular adverse events (CVAEs) according to CTCAE version 5.0; Table S3: Absolute cardiac biomarker levels by cardiovascular event status; Table S4: Cardiovascular treatment at baseline, by cardiovascular event status; Table S5: Cardiovascular Adverse Events (CVAEs) by Grade and HFA-ICOS Risk Category.
