# Supplementary material for: Validation and Exploratory Refinement of the HFA-ICOS Score for Cardiovascular Risk in Proteasome Inhibitor-Treated Multiple Myeloma: Single-Center Retrospective Study
Source: Cancers (Basel). 2026 Jun 12;18(12):1924. doi: 10.3390/cancers18121924 (PMC13297542; doi:10.3390/cancers18121924)
Supplement: Supplementary file 1 [file cancers-18-01924-s001.zip › Supplementary Table S1..pdf]

Risk factors and assigned risk levels according to the original HFA-ICOS score are shown below. Criteria used to derive overall risk categories are summarized at the end of the table.

Supplementary Table S1. Baseline cardiovascular risk stratification scale for proteasome inhibitors and immunomodulatory agents for multiple myeloma

**Risk Factor**	**Risk Category**	**Level of Evidence**
**Previous cardiovascular disease**		
Heart failure or cardiomyopathy	VERY HIGH	C
Prior proteasome inhibitor cardiotoxicity	VERY HIGH	C
Venous thrombosis (DVT or PE)	VERY HIGH	C
Cardiac amyloidosis	VERY HIGH	C
Arterial vascular disease (IHD, PCI, CABG, stable angina, TIA, stroke, PVD)	VERY HIGH	C
Prior immunomodulatory drug (IMiD) CV toxicity	HIGH	B
Baseline LVEF <50%	HIGH	C
Borderline LVEF 50–54%	MEDIUM[2]	C
Arrhythmia*	MEDIUM[2]	C
Left ventricular hypertrophy**	MEDIUM[1]	C
Cardiac biomarkers (where available)		
Elevated baseline troponin***	MEDIUM[2]	C
Elevated baseline BNP or NT-proBNP***	HIGH	B
**Demographic and CV risk factors**		
Age ≥75 years	HIGH	C
Age 65–74 years	MEDIUM[1]	C
Hypertension	MEDIUM[1]	C
Diabetes mellitus†	MEDIUM[1]	C
Hyperlipidemia††	MEDIUM[1]	C
Chronic kidney disease▲	MEDIUM[1]	C

Family history of thrombophilia	MEDIUM[1]	C
**Previous cardiotoxic cancer treatment**		
Prior anthracycline exposure	HIGH	C
Prior thoracic spine radiotherapy	MEDIUM[1]	C
**Current cancer treatment**		
High-dose dexamethasone ≥160 mg/month	MEDIUM[1]	C
**Lifestyle risk factors**		
Current smoker or significant smoking history	MEDIUM[1]	C
Obesity (BMI >30)	MEDIUM[1]	C

<u>Risk Category Definitions:</u>

Low risk = no risk factor or one medium[1] risk factor

Medium risk = medium risk factors with a total of 2–4 points

High risk = total of ≥5 points from medium* or one high risk factor

Very high risk = any very high risk factor

BMI = Body mass index; BNP = Brain natriuretic peptide; CABG = Coronary artery bypass graft; DVT = Deep vein thrombosis; IHD = Ischaemic heart disease; LVEF = Left ventricular ejection fraction; NT-proBNP = N-terminal pro–B-type natriuretic peptide; PCI = Percutaneous coronary intervention; PE = Pulmonary embolism; PVD = Peripheral vascular disease; TIA = Transient ischemic attack

* Atrial fibrillation, atrial flutter, ventricular tachycardia, or ventricular fibrillation

** Left ventricular hypertrophy on echocardiogram (septal wall thickness ≥12 mm)

*** Elevated above the upper limit of normal for the local laboratory reference range

† Systolic blood pressure ≥140 mmHg or diastolic ≥90 mmHg, or on antihypertensive treatment

†† Total non-HDL cholesterol ≥3.8 mmol/L (≥145 mg/dL)

▲ Estimated glomerular filtration rate <60 mL/min/1.73m^2
