# Supplementary material for: Validation and Exploratory Refinement of the HFA-ICOS Score for Cardiovascular Risk in Proteasome Inhibitor-Treated Multiple Myeloma: Single-Center Retrospective Study
Source: Cancers (Basel). 2026 Jun 12;18(12):1924. doi: 10.3390/cancers18121924 (PMC13297542; doi:10.3390/cancers18121924)
Supplement: Supplementary file 1 [file cancers-18-01924-s001.zip › Supplementary Table S2..pdf]

Supplementary Table S2. Definitions and grading of cardiovascular adverse events (CVAEs) according to CTCAE version 5.0

Adverse Event	Grade 1	Grade 2	Grade 3	Grade 4	Grade 5
**Arrhythmia (AF/flutter)**	Asymptomatic; intervention not indicated	Symptomatic; non-urgent medical intervention indicated	Severe symptoms; hospitalization indicated; medically significant but not immediately life-threatening	Life-threatening consequences; urgent intervention indicated	Death
**Heart Failure (HF)**	Asymptomatic; mild objective findings only	Mild symptoms (e.g., exertional dyspnea) with minimal intervention needed	Symptomatic; limiting activities of daily living; hospitalization indicated	Life-threatening (e.g., severe pulmonary edema, cardiogenic shock)	Death
**Hypertension (HTN)**	Prehypertension; transient; no intervention required	Systolic BP ≥140–159 mmHg or diastolic BP ≥90–99 mmHg; medical intervention indicated	Systolic BP ≥160 mmHg or diastolic ≥100 mmHg; hospitalization indicated; urgent intervention required	Life-threatening (e.g., malignant hypertension, hypertensive crisis, neurologic/cardiac compromise)	Death
**Ischemic Events (MI, Stroke, TIA)**	Asymptomatic, mild lab/imaging findings only	Symptomatic but not urgent (e.g., stable	Severe symptoms; hospitalization required (e.g.,	Life-threatening (e.g., STEMI, disabling stroke, ischemia with hemodynamic compromise)	Death due to ischemia

		angina, TIA without deficit)	non-STEMI, ischemic stroke with recovery)		
**Thromboembolic Events (DVT, PE)**	Asymptomatic incidental finding (e.g., small DVT on imaging)	Symptomatic but not urgent; medical intervention indicated (e.g., superficial thrombosis)	Severe symptoms; hospitalization required (e.g., PE requiring anticoagulation, extensive DVT)	Life-threatening (e.g., massive PE with shock, systemic embolism with compromise)	Death due to thrombosis/embolism
**Cardiovascular Death (CVD)**	–	–	–	–	Death directly attributable to cardiovascular cause (e.g., MI, HF, arrhythmia, stroke, embolism)

Abbreviations: AF = atrial fibrillation; HF = heart failure; HTN = hypertension; BP = blood pressure; MI = myocardial infarction; TIA = transient ischemic attack; DVT = deep vein thrombosis; PE = pulmonary embolism; STEMI = ST-elevation myocardial infarction; CVD = cardiovascular death.
