# Supplementary material for: Validation and Exploratory Refinement of the HFA-ICOS Score for Cardiovascular Risk in Proteasome Inhibitor-Treated Multiple Myeloma: Single-Center Retrospective Study
Source: Cancers (Basel). 2026 Jun 12;18(12):1924. doi: 10.3390/cancers18121924 (PMC13297542; doi:10.3390/cancers18121924)
Supplement: Supplementary file 1 [file cancers-18-01924-s001.zip › Supplementary table S3..pdf]

Supplementary Table S3. Absolute cardiac biomarker levels by cardiovascular event status

	All patients (n=98)	Cardiovascular Events (n=22)	Non-Cardiovascular Events (n=78)	p
NT-ProBNP, *mean ± SEM*	1740 ± 698	2612 ± 1192	1479 ± 836	0.073
Pre-cycle 2 NT-ProBNP, *mean ± SEM*	1240 ± 467	479 ± 105	3872 ± 1981	**0.018**
hs-TnT, *mean ± SEM*	27.7 ± 4.8	38.5 ± 8.3	25.4 ± 5.8	0.093

Abbreviations: NT-ProBNP: N-terminal pro–B-type natriuretic peptide; SEM: Standard Error of the mean; Tropt-T-US: High-sensitivity Troponin T
