# Supplementary material for: Validation and Exploratory Refinement of the HFA-ICOS Score for Cardiovascular Risk in Proteasome Inhibitor-Treated Multiple Myeloma: Single-Center Retrospective Study
Source: Cancers (Basel). 2026 Jun 12;18(12):1924. doi: 10.3390/cancers18121924 (PMC13297542; doi:10.3390/cancers18121924)
Supplement: Supplementary file 1 [file cancers-18-01924-s001.zip › Supplementary table S4..pdf]

Supplementary Table S4. Cardiovascular treatment at baseline, by cardiovascular event status

	All patients (n=98)	Cardiovascular Events (n=22)	Non-Cardiovascular Events (n=78)	p
Beta-blockers	22 (22.4)	10 (45.5)	12 (15.8)	**0.003**
ACE inhibitors/ARBs	41 (41.8)	13 (59.1)	28 (36.8)	0.062
SGLT2 inhibitors	3 (3.1)	0 (0.0)	3 (3.9)	0.34
Anticoagulants	71 (72.4)	14 (63.3)	57 (75.0)	0.29
Aldosterone Antagonists	1 (1.0)	0 (0.0)	1 (1.3)	0.59
Calcium channel blockers	18 (18.4)	9 (40.9)	9 (11.8)	**0.002**
Antiplatelet agents	27 (27.6)	12 (54.5)	15 (19.7)	**0.001**
Antiarrythmics	0 (0.0)	0 (0.0)	0 (0.0)	0.99
Diuretics	27 (27.6)	9 (40.9)	18 (23.7)	0.11
