# Supplementary material for: Validation and Exploratory Refinement of the HFA-ICOS Score for Cardiovascular Risk in Proteasome Inhibitor-Treated Multiple Myeloma: Single-Center Retrospective Study
Source: Cancers (Basel). 2026 Jun 12;18(12):1924. doi: 10.3390/cancers18121924 (PMC13297542; doi:10.3390/cancers18121924)
Supplement: Supplementary file 1 [file cancers-18-01924-s001.zip › Supplementary Table S5..pdf]

Supplementary Table S5. Cardiovascular Adverse Events (CVAEs) by Grade and HFA-ICOS Risk Category

Cardiovascular adverse event by grade N (%)	All patients (n=98)	Low Risk (n=10)	Medium Risk (n=13)	High Risk (n=49)	Very High Risk (n=26)
**Heart failure**					
1-2	6 (6.1%)	0 (0.0%)	0 (0.0%)	0 (0.0%)	6 (23.1%)
3-4	4 (4.1%)	1 (10.0%)	0 (0.0%)	1 (2.0%)	2 (7.7%)
**All**	10 (10.2%)	1 (10.0%)	0 (0.0%)	1 (2.0%)	8 (30.8%)
**Arrhythmia**					
1-2	6 (6.1%)	0 (0.0%)	0 (0.0%)	4 (8.2%)	2 (7.7%)
3-4	0 (0.0%)	0 (0.0%)	0 (0.0%)	0 (0.0%)	0 (0.0%)
**All**	6 (6.1%)	0 (0.0%)	0 (0.0%)	4 (8.2%)	2 (7.7%)
**Hypertension**					
1-2	3 (3.1%)	0 (0.0%)	0 (0.0%)	1 (2.0%)	2 (7.7%)
3-4	4 (4.1%)	0 (0.0%)	1 (7.7%)	3 (6.1%)	0 (0.0%)
**All**	7 (7.2%)	0 (0.0%)	1 (7.7%)	4 (8.2%)	2 (7.7%)
**Ischemic events**					
1-2	0 (0.0%)	0 (0.0%)	0 (0.0%)	0 (0.0%)	0 (0.0%)
3-4	1 (1.0%)	0 (0.0%)	0 (0.0%)	0 (0.0%)	1 (3.8%)
**All**	1 (1.0%)	0 (0.0%)	0 (0.0%)	0 (0.0%)	1 (3.8%)
**Thromboembolic events**					
1-2	1 (1.0%)	0 (0.0%)	0 (0.0%)	0 (0.0%)	1 (3.8%)
3-4	1 (1.0%)	0 (0.0%)	1 (7.7%)	0 (0.0%)	0 (0.0%)
**All**	2 (2.0%)	0 (0.0%)	1 (7.7%)	0 (0.0%)	1 (3.8%)
**Total**					
1-2	16 (16.3%)	0 (0.0%)	0 (0.0%)	5 (10.2%)	11 (42.3%)
3-4	10 (10.2%)	1 (10.0%)	2 (15.4%)	4 (8.2%)	3 (11.5%)
**All**	26 (26.5%)	1 (10.0%)	2 (15.4%)	9 (18.4%)	14 (53.8%)
