# Supplementary figures and images for: Validation and Exploratory Refinement of the HFA-ICOS Score for Cardiovascular Risk in Proteasome Inhibitor-Treated Multiple Myeloma: Single-Center Retrospective Study
Source: Cancers (Basel). 2026 Jun 12;18(12):1924. doi: 10.3390/cancers18121924 (PMC13297542; doi:10.3390/cancers18121924)

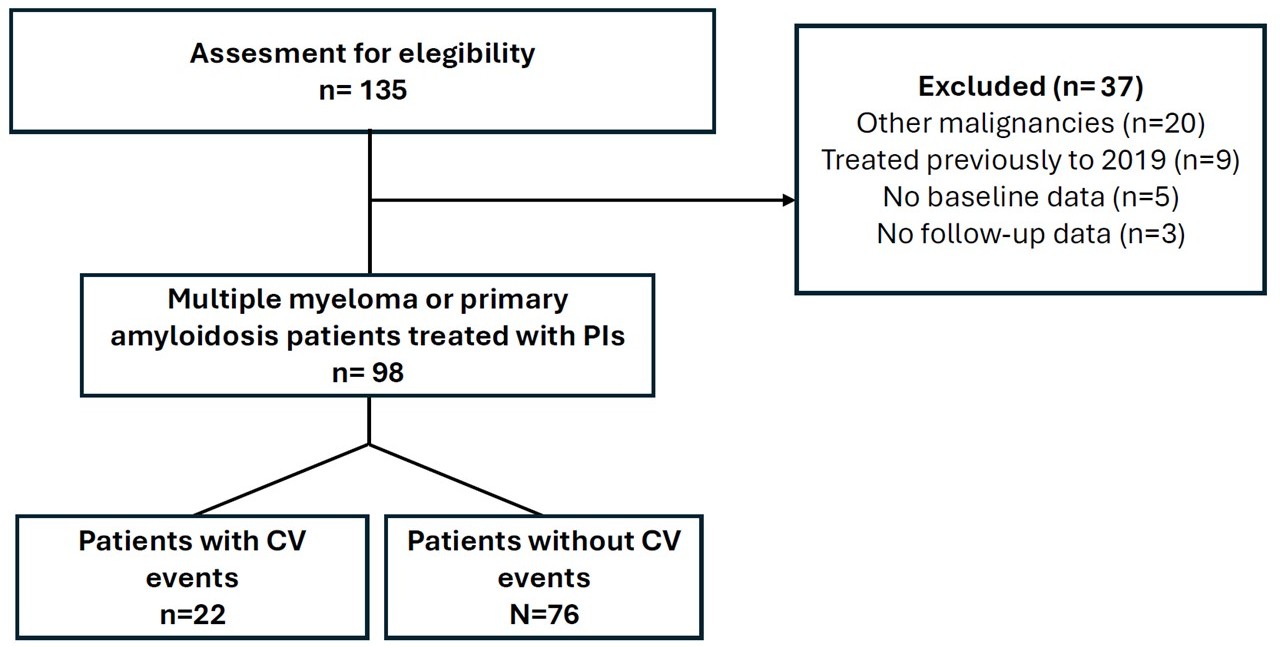

Supplement: Supplementary file 1 [file cancers-18-01924-s001.zip › Supplementary Figure S1-Flow diagram.jpg]
